# Efficacy and specific toxicity profiles of GPRC5D- versus BCMA-targeted bispecific antibodies in relapsed/refractory multiple myeloma: a meta-analysis of clinical trials and real-world studies

**DOI:** 10.3389/fimmu.2026.1937154

**Published:** 2026-09-29

**Authors:** Jiahao Ji, Xinyu Zhao, Bin Wang, Jiayu Li, Fangliang Zhou

**Affiliations:** 1 Medical School, Hunan University of Chinese Medicine, Changsha, China; 2 Acupuncture, Tuina and Rehabilitation College, Hunan University of Chinese Medicine, Changsha, China

**Keywords:** BCMA×CD3, bispecific antibody, epithelium-related adverse events, GPRC5D×CD3, hematological toxicity, relapsed/refractory multiple myeloma

## Abstract

**Background:**

Patients with relapsed/refractory multiple myeloma (RRMM) experience poor outcomes following multiple lines of therapy. Available evidence has largely pooled disparate immunotherapy platforms. The present systematic review and meta-analysis was performed to describe, separately by target, the efficacy and toxicity spectra of BCMA×CD3-directed, GPRC5D×CD3-directed, and dual-target bispecific antibody therapy in RRMM, in order to inform toxicity monitoring and supportive-care planning.

**Methods:**

The Cochrane Library, Embase, Web of Science, and PubMed were systematically searched up to January 28, 2026, to identify clinical trials and real-world studies evaluating GPRC5D×CD3 or BCMA×CD3 bispecific antibodies and their combinations for RRMM. Methodological quality was appraised with the Methodological Index for Non-Randomized Studies. Proportions and 95% confidence intervals (CIs) were pooled utilizing fixed-effects or random-effects models in R, selected according to heterogeneity.

**Results:**

Twenty-seven studies—comprising 13 single-arm clinical trials and 14 real-world investigations involving 4,045 patients—met the inclusion criteria. The pooled objective response rate (ORR) for bispecific antibodies in RRMM was 65.54%. Target-stratified subgroup analysis yielded an ORR of 78.51% for GPRC5D×CD3 combined with BCMA×CD3, 69.67% for GPRC5D×CD3 alone, and 60.85% for BCMA×CD3 alone. The pooled minimal residual disease-negativity rate was 23.6%. Regarding safety, distinct antigen-specific toxicity spectra emerged across different targets. The BCMA×CD3 group exhibited elevated incidences of any-grade infection (56.37%) and grade 3–4 infection (26.37%), accompanied by pronounced hematological toxicity. The GPRC5D×CD3 group was characterized by epithelium-related toxicity: the incidence of grade 1–2 taste-related changes was 69.89%, that of any-grade nail-related adverse events (AEs) was 42.33%, and that of any-grade non-rash skin-related AEs was 58.3%; however, grade 3–4 nail-related AEs reached only 0.21%, and grade 3–4 non-rash skin-related AEs reached only 0.33%. The dual-target group bore a substantial burden of infection (84.53%) and hematological toxicity.

**Conclusion:**

Bispecific antibodies demonstrate clear antimyeloma activity in RRMM. BCMA×CD3-directed therapy is mainly related to infection and hematological toxicity, whereas GPRC5D×CD3-directed therapy is distinguished by epithelium-related toxicity, although severe events are rare.

**Systematic review registration:**

https://www.crd.york.ac.uk/PROSPERO/ identifier, CRD420261296139.

## 1 Introduction

Multiple myeloma (MM) is a clonal plasma-cell malignancy that remains largely incurable. Most patients eventually develop relapsed/refractory multiple myeloma (RRMM) after exposure to proteasome inhibitors, immunomodulatory drugs, and anti-CD38 monoclonal antibodies (1, 2). An estimated 188,000 new cases and more than 120,000 deaths were reported worldwide in 2022. RRMM is also associated with a substantial burden of recurrent relapses, infections, hospitalizations, and treatment-related costs. Optimizing treatment sequencing while minimizing therapy-related toxicity has thus become a central challenge in the management of RRMM (3–5).

T-cell-redirecting bispecific antibodies have rapidly reshaped this landscape by simultaneously engaging CD3^+^ T cells and plasma-cell antigens, principally B-cell maturation antigen (BCMA) and G protein-coupled receptor class C group 5 member D (GPRC5D) (6). BCMA is expressed on both normal and malignant plasma cells. BCMA-directed agents are typically associated with cytokine release syndrome (CRS), cytopenias, immune effector cell-associated neurotoxicity syndrome (ICANS), and clinically significant infections (7–9). GPRC5D is largely restricted to malignant plasma cells but is also expressed in skin, nails, hair follicles, and taste buds, rendering talquetamab characteristically prone to on-target/off-tumor taste changes and skin and nail disorders (10–12). Given fundamental differences in the biological substrates of these toxicities, pooling distinct targets obscures the true burden of target-specific adverse events (AEs). A dual-target strategy combining GPRC5D×CD3 with BCMA×CD3 has additionally emerged, while its superimposed toxicity remains unquantified by synthesized evidence (13).

Although bispecific antibodies have rapidly reshaped the treatment landscape of RRMM, the synthesis of available evidence remains insufficient to directly guide bedside therapeutic decisions (14). Prior meta-analyses have predominantly concentrated on BCMA-directed therapy or have aggregated disparate immunotherapy platforms—including chimeric antigen receptor (CAR) T-cell therapy, antibody–drug conjugates, and bispecific antibodies—thereby obscuring the target-specific toxicity signals inherent to distinct antigens (15, 16). A recent investigation incorporates both BCMA×CD3 and GPRC5D×CD3 bispecific antibodies. Still, that study does not evaluate the dual-target combination of GPRC5D×CD3 plus BCMA×CD3 as an independent strategy, nor does it systematically include minimal residual disease (MRD) negativity, a pivotal endpoint reflecting depth of remission (17). Through a systematic review and target-stratified meta-analysis, the present research quantitatively characterized the efficacy—encompassing MRD negativity—and antigen-specific toxicity spectra of three bispecific antibody strategies: BCMA×CD3, GPRC5D×CD3, and their combination in RRMM. Given that all available evidence is single-arm, the objective was explicitly descriptive: to define the characteristic toxicity signature of each target and translate it into monitoring and supportive-care strategies, not to establish comparative superiority between targets.

## 2 Methods

The current systematic review and meta-analysis was conducted per the Preferred Reporting Items for Systematic Reviews and Meta-Analyses (PRISMA) guidelines (18). Its protocol was prospectively registered with the International Prospective Register of Systematic Reviews (PROSPERO; CRD420261296139).

### 2.1 Data source and search strategy

Ji and Zhao independently searched the Cochrane Library, Web of Science, Embase, and PubMed up to January 28, 2026. The search strategy employed Boolean operators to link various search terms, incorporating both medical subject headings and free-text terms: ‘multiple myeloma’, ‘BCMA’, ‘GPRC5D’, and ‘bispecific antibody’. To avoid omission, the reference lists of the eligible studies were also hand-searched for additional investigations not identified in the initial search. Any discrepancy between reviewers was resolved through consultation with FangLiang Zhou. The detailed search strategy for each database is available in **Supplementary Table 1**.

### 2.2 Eligibility criteria

Studies meeting the following criteria were eligible for inclusion.

P (Population): RRMM.

I (Intervention): bispecific antibodies targeting GPRC5D, BCMA, or their combination.

C (Comparison): The present investigation did not require a control group; studies of the following types were eligible for inclusion: single-arm clinical trials or real-world studies lacking a comparator, used to pool the incidence of toxicity across various targeted bispecific antibodies; where a study included other treatment modalities as a control arm, only data pertinent to GPRC5D- or BCMA-directed bispecific antibodies were extracted.

O (Outcomes): overall response rate (ORR), partial response (PR), very good partial response (VGPR), complete response (CR), stringent complete response (sCR), MRD negativity, hematological toxicity, infection, CRS, ICANS, diarrhea, skin disorders, taste-related changes or taste disturbance, and nail-related AEs.

S (Study design): prospective or retrospective clinical trials, including phase I, phase I/II, phase II, or phase III studies; randomized or non-randomized controlled studies; single-arm interventional studies; and real-world investigations, including retrospective cohort studies, prospective cohort studies, and multicenter observational studies.

Reports meeting the following conditions were excluded: (i) duplicate publications; (ii) non-original research (including reviews, case reports, conference abstracts, commentaries, study protocols, and clinical guidelines); (iii) *in vitro* or animal studies; (iv) non-English-language publications; (v) reports with missing or critically erroneous data.

### 2.3 Study selection

All retrieved records were imported into EndNote 21. After removing duplicates, Ji and Zhao independently screened all possibly relevant studies by reviewing titles and abstracts. Subsequently, full texts of potentially eligible articles were evaluated against the predefined eligibility criteria. Twenty-seven single-arm clinical trials and real-world studies were ultimately included. Any discrepancies were resolved through discussion with FangLiang Zhou.

### 2.4 Data extraction

Ji and Zhao independently selected the studies and extracted the data to minimize bias from the extraction by a single individual. When consensus could not be reached, FangLiang Zhou resolved any discrepancies. Upon confirmation of eligible studies, the following data were extracted from each study into an Excel spreadsheet: basic information (first author, study design, publication year, study phase, sample size), baseline characteristics of patients (age, disease burden, sex, high-risk cytogenetics, extramedullary disease, International Staging System stage, Eastern Cooperative Oncology Group performance status), prior treatment history (number of prior lines of therapy, prior transplantation of hematopoietic stem cells, triple-class exposure/refractoriness, penta-drug exposure/refractoriness, refractoriness to the last line of therapy), treatment characteristics (target, agent name, dosing schedule), and efficacy and safety outcomes.

### 2.5 Risk of bias assessment

Ji and Zhao independently appraised the quality of eligible studies. Design-specific instruments were adopted: the methodological quality of non-randomized studies was evaluated using the 12-item Methodological Index for Non-Randomized Studies (MINORS) tool (19). Each item receives a score of 0 (not reported), 1 (reported with insufficient detail), or 2 (reported with adequate detail). Higher aggregate scores reflect superior methodological quality, with a maximum score of 16 for non-comparative studies and 24 for comparative studies. Disagreements in appraisal were settled through consultation with FangLiang Zhou.

### 2.6 Statistical analysis

For the statistical analysis, the total number of patients in each original study was first recorded, and the number of individuals experiencing each outcome of interest was then extracted. Meta-analyses were completed utilizing the R statistical software to derive the pooled incidence and corresponding 95% confidence interval (CI) for each outcome in patients with RRMM. Heterogeneity across studies was evaluated by computing the I² statistic. I² values of 25%, 50%, and 75% were taken to represent low, moderate, and high degrees of heterogeneity, respectively. A fixed-effects model was applied when heterogeneity was low to moderate (I² ≤ 50%); otherwise, a random-effects model was adopted (20). Leave-one-out sensitivity analysis was carried out to identify whether any single study exerted undue influence on the overall result (21). Publication bias was examined via funnel plots when more than ten studies were available for a given outcome; otherwise, such evaluation was not conducted, as the statistical power would be too low to distinguish genuine asymmetry from chance. For all outcome variables, a two-tailed *p*-value < 0.05 was considered statistically significant. All statistical analyses were carried out in R (v4.5.0).

## 3 Results

### 3.1 Literature screening

The systematic search yielded 14,805 records across 4 databases. After removing 2,638 duplicates using EndNote 21, 12,167 studies were retained for screening. After the initial screening, full texts of 68 possibly relevant studies were retrieved and reviewed, of which 41 were removed due to inconsistency with the study purpose (n = 29), ineligible population (n = 2), ongoing trials (n = 5), mismatched intervention (n = 1), and mismatched outcome indicators (n = 4). Ultimately, 27 (22–48) studies were eligible for inclusion (**Figure 1**).

**Figure 1:**
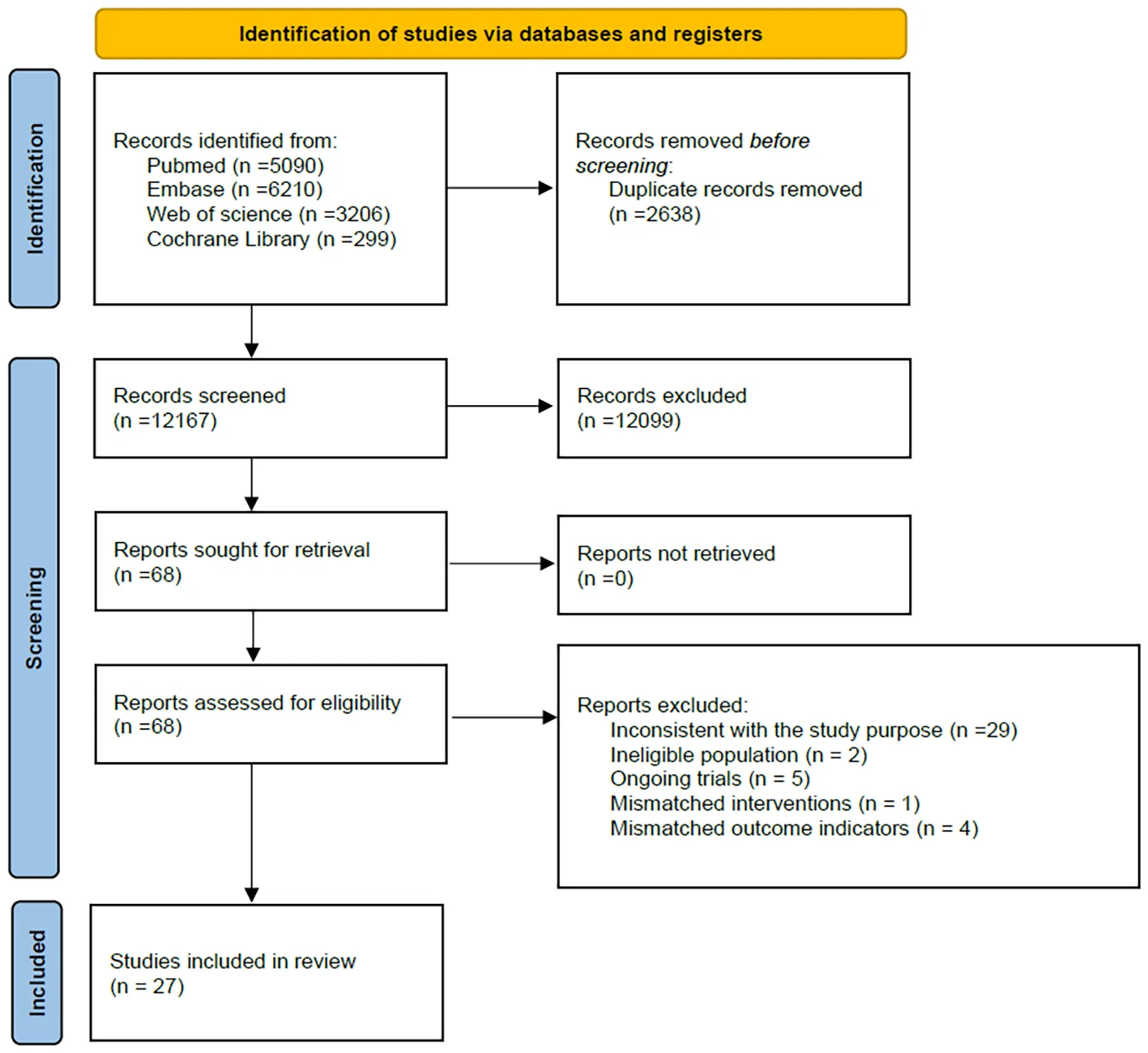
PRISMA 2020 flowchart, step-by-step screening process and selection of studies.

**Table 1** summarizes the baseline characteristics of 13 single-arm clinical trials encompassing 21 treatment cohorts (1,708 patients with RRMM), with study phases ranging from phase I to phase II. Regarding the target, 9 cohorts evaluated GPRC5D×CD3 (talquetamab), 9 evaluated BCMA×CD3 (teclistamab, elranatamab, linvoseltamab), and a further 3 evaluated GPRC5D×CD3 in combination with BCMA×CD3 (talquetamab plus teclistamab). Sample sizes of these cohorts ranged from 4 to 165 individuals, with median ages spanning 61 to 70.5 years; the populations were predominantly White. The proportion of patients harboring high-risk cytogenetic abnormalities ranged from 11% to 73%. The median number of prior lines of therapy varied from 3.5 to 7.5. Triple-class-refractory disease was present in 65.4% to 100% of patients, and penta-drug-refractory disease was present in 20% to 58.3% of patients. Detailed baseline characteristics for each cohort are presented in **Supplementary Table 2**.

**Table 1:** Baseline characteristics of 13 single-arm clinical trials.

Study	Phase	BsAb target	Drug name	n	Age median	High-risk cytogenetic abnormalities	Previous lines of therapy	Triple-class refractory	Penta-drug refractory
MonumenTAL-1 (S/C QW arm) Chari et al,(2025) (22)	1–2 study	GPRC5D × CD3	Talquetamab	143	67 (58–72)	41/132 (31%)	5·0 (4·0–6·0)	107 (75%)	45 (31%)
MonumenTAL-1 (S/C Q2W arm) Chari et al,(2025) (22)	1–2 study	GPRC5D × CD3	Talquetamab	154	67 (58–74)	40/133 (30%)	4·5 (4·0–6·0)	110 (71%)	39 (25%)
MonumenTAL-1 (Patients with previous TCR arm) Chari et al,(2025) (22)	1–2 study	GPRC5D × CD3	Talquetamab	78	61 (55–68)	25/67 (37%)	6·0 (5·0–8·0)	66 (85%)	34 (44%)
MonumenTAL-1 (S/C QW arm) Chari et al,(2022) (23)	1–2 study	GPRC5D × CD3	Talquetamab	30	62 (46–80)	3/27 (11%)	6 (2–14)	23 (77%)	6 (20%)
MonumenTAL-1 (S/C Q2W arm) Chari et al,(2022) (23)	1–2 study	GPRC5D × CD3	Talquetamab	44	64 (47–84)	9/40 (22%)	5 (2–17)	33 (75%)	9 (20%)
MonumenTAL-1 (Subcutaneous arm) Chari et al,(2022) (23)	1–2 study	GPRC5D × CD3	Talquetamab	130	64 (39–84)	18/112 (16%)	6 (2–17)	97 (75%)	33 (25%)
MonumenTAL-1 (Intravenous arm) Chari et al,(2022) (23)	1–2 study	GPRC5D × CD3	Talquetamab	102	65 (33–79)	14/88 (16%)	6 (3–20)	87 (85%)	36 (35%)
MonumenTAL-1 (S/C QW arm) Ito et al,(2025) (24)	1–2 study	GPRC5D × CD3	Talquetamab	36	70.5 (46–81)	13/36(36%)	3.5 (3–13)	25 (69.4%)	8 (22.2%)
Phase 1 study of Japanese patients (Subcutaneous arm) Iida et al,(2025) (25)	1 study	GPRC5D × CD3	Talquetamab	15	65.0 (45–86)	11/15 (73%)	5.0 (2–16)	12 (80.0%)	6 (40.0%)
MajesTEC-1 Moreau et al. (2022) (26)	1–2 study	BCMA × CD3	Teclistamab	165	64.0(33–84)	38/148 (25.7%)	5 (2–14)	128 (77.6%)	50 (30.3%)
MagnetisMM-3 Lesokhin et al. (2023) (27)	2 study	BCMA × CD3	Elranatamab	123	68.0 (36–89)	31 (25.2%)	5 (2–22)	119 (96.7%)	52 (42.3%)
MajesTEC-1 Usmani et al. (2021) (28)	1 study	BCMA × CD3	Teclistamab	157	63 (57–69)	38 (33%)	6 (4–7)	128 (82%)	61 (39%)
LINKER-MM1 (50mg Intravenous arm) Bumma et al. (2024) (29)	1–2 study	BCMA × CD3	Linvoseltamab	104	65 (45-90)	28 (26.9%)	6 (3-14)	97(93.3%)	56(53.8%)
LINKER-MM1 (200mg Intravenous arm) Bumma et al. (2024) (29)	1–2 study	BCMA × CD3	Linvoseltamab	117	70 (37-91)	46 (39.3%)	5 (2-16)	96(82.1%)	33(28.2%)
MajesTEC-1 Touzeau et al. (2024) (30)	1–2 study	BCMA × CD3	Teclistamab	40	63.5 (32-82)	12 (30.0%)	6 (3-14)	34 (85.0%)	14 (35.0%)
NCT04696809 Ishida et al. (2024) (31)	1–2 study	BCMA × CD3	Teclistamab	26	67.5	5 (19.2%)	4.5 (3–12)	17 (65.4%)	6 (23.1%)
MagnetisMM-2 Iida et al. (2024) (32)	1 study	BCMA × CD3	Elranatamab	4	68.5 (49–70)	1 (25.0%)	7.5 (6-9)	4(100.0%)	1(25%)
MagnetisMM-3 Iida et al. (2024) (32)	2 study	BCMA × CD3	Elranatamab	12	66.5 (47–83)	5 (41.7%)	6.0(2-11)	12(100.0%)	7(58.3%)
RedirecTT-1 (RP2R arm) Cohen et al. (2025) (33)	1b–2 study	GPRC5D × CD3 + BCMA × CD3	Talquetamab+Teclistamab	44	63 (41–80)	8/19 (42%)	4 (2–10)	37 (84%)	13 (30%)
RedirecTT-1 (All Dose Levels arm) Cohen et al. (2025) (33)	1b–2 study	GPRC5D × CD3 + BCMA × CD3	Talquetamab+Teclistamab	94	64.5 (39–81)	21/51 (41%)	4 (1–11)	81 (86%)	31 (33%)
RedirecTT-1 (RP2R arm) Kumar et al. (2025) (34)	2 study	GPRC5D × CD3 + BCMA × CD3	Talquetamab+Teclistamab	90	64.5 (42–84)	14/65 (22%)	4 (1–10)	76(84.4%)	32(35.6%)

**Table 2** summarizes the baseline characteristics of 14 real-world investigations encompassing 18 cohorts (2,337 patients), with study designs including single-center, multicenter retrospective/prospective observational studies, and international multicenter studies. Regarding the target, 4 cohorts evaluated GPRC5D×CD3 (predominantly talquetamab), 13 evaluated BCMA×CD3 (teclistamab, elranatamab, or a combination thereof), and 1 cohort comprised patients treated with both classes of bispecific antibodies. Sample sizes of these cohorts ranged from 18 to 509 individuals, with median ages spanning 60 to 78 years; the populations were primarily White. The proportion of patients harboring high-risk cytogenetic abnormalities ranged from 23.8% to 70%. The median number of prior lines of therapy varied from 4 to 7. Triple-class-refractory disease was present in 40.5% to 100% of patients, and penta-drug-refractory disease was present in 19% to 88.9% of patients. Detailed baseline characteristics for each cohort are provided in **Supplementary Table 3**.

**Table 2:** Baseline characteristics of 14 real-world studies.

Study	Phase	BsAb target	Drug name	n	Age median	High-risk cytogenetic abnormalities	Previous lines of therapy	Triple-class refractory	Penta-drug refractory
Winship Cancer Institute of Emory University. (GPRC5D-targeting BsAb arm) Cani et al,(2025) (35)	single-center experience	GPRC5DxCD3	Talquetamab	57	65 (38-89)	NA	6 (3-16)	51 (89%)	NA
a multicenter, prospective, observational study at the University Hospitals Würzburg and Heidelberg (GPRC5D-group arm) Fleischer et al,(2025) (36)	Real-World Experience	GPRC5DxCD3	Talquetamab	26	64 (48-85)	NA	NA	NA	NA
A German multicenter real‐world analysis (Real‐world cohort) Frenking et al,(2025) (37)	Real-World Experience	GPRC5DxCD3	Talquetamab	138	64 (24-84)	57/120 (48%)	6 (2-15)	118/138 (86%)	64/136 (47%)
Four academic institutions in the United States Hadidi et al,(2025) (38)	multicenter, retrospective study	GPRC5DxCD3	Talquetamab	114	67 (34-90)	64 (70%)	6 (2-15)	114 (100%)	90 (79%)
multi-institutional real-world study Mohan et al,(2024) (39)	Real-World Experience	BCMA×CD3	Teclistamab	110	68 (37-89)	59/95 (62%)	6 (3-13)	95 (86%)	84 (76%)
a multicenter study from the U.S. (Age ≥75 arm) Pasvolsky et al,(2025) (40)	Real-World Experience	BCMA×CD3	Teclistamab	83	78 (75-92)	37 (45%)	NA	63 (77%)	24 (30%)
A multicenter study from the U.S. (Age <75 arm) Pasvolsky et al,(2025) (40)	Real-World Experience	BCMA×CD3	Teclistamab	302	65 (31-74)	175 (58%)	NA	257 (85%)	118 (39%)
A multicenter study from the U.S. Razzo et al,(2025) (41)	Real-World Experience	BCMA×CD3	Teclistamab	509	68 (31-92)	275 (54%)	6 (2-18)	405/500 (81%)	192/500 (38%)
Czech myeloma group real-world evidence analysis (Non-weekly dosing arm) Stork et al,(2025) (42)	Real-World Experience	BCMA×CD3	Teclistamab	18	69 (49-83)	NA	4 (3-9)	NA	10(55.6%)
Czech myeloma group real-world evidence analysis (Weekly dosing arm) Stork et al,(2025) (42)	Real-World Experience	BCMA×CD3	Teclistamab	55	66 (41-85)	NA	5 (3-13)	NA	40(88.9%)
an International Myeloma Working Group Study Tan et al,(2025) (43)	Real-World Experience	BCMA×CD3	Teclistamab	210	67 (33-91)	82/164 (50%)	6 (1-20)	138/167 (82.6%)	71/161 (44.1%)
Nationwide Retrospective Analysis of the Named Patient Program in Korea Yi et al,(2024) (44)	Real-World Experience	BCMA×CD3	Teclistamab	42	60 (43-80)	10(23.8%)	NA	17 (40.5%)	8 (19.0%)
Multicenter study of 5 large US academic centers Dima et al,(2023) (45)	Real-World Experience	BCMA×CD3	Teclistamab	106	66.5 (35-87)	56/95 (59%)	6 (4-17)	97 (92)%	68 (64%)
Data were collected from 13 academic centers participating in the US MM Immunotherapy Consortium. (No RI arm) Dima et al,(2025) (46)	Real-World Experience	BCMA×CD3	Teclistamab	303	67 (60.2-72.1	164 (54%)	6 (5-8)	257 (85%)	113 (37%)
a multicenter, prospective, observational study at the University Hospitals Würzburg and Heidelberg (BCMA-group arm) Fleischer et al,(2025) (36)	Real-World Experience	BCMA×CD3	Teclistamab or Elranatamab	26	64.5 (52-79)	NA	NA	NA	NA
Winship Cancer Institute of Emory University. (BCMA-targeting BsAb arm) Cani et al,(2025) (35)	single-center experience	BCMA×CD3	Teclistamab or Elranatamab	101	66 (43-87)	NA	5 (2-14)	80 (79%)	NA
Real-world evidence from Sweden Uttervall et al,(2024) (47)	Real-World Experience	BCMA×CD3	Teclistamab or Elranatamab	58	62.9 (37-80)	15 (26%)	7 (3-17)	52 (90%)	41 (71%)
John Theurer Cancer Center (All samples) Fogel et al,(2025) (48)	single center experience	GPRC5DxCD3 or BCMA×CD3	Talquetamab or Teclistamab/ Elranatamab	79	68 (59.5-77)	36 (46%)	5 (2-18)	79 (100%)	19 (24%)

### 3.2 Risk of bias evaluation

Quality appraisal using the MINORS instrument indicated that 10 studies were of high quality and 17 were of moderate quality. A comprehensive summary of the evaluation is available in **Supplementary Table 4**.

### 3.3 Efficacy

#### 3.3.1 Incidence of ORR

Twenty-four studies reported ORR following treatment with bispecific antibodies for RRMM, encompassing 3,623 patients. Meta-analysis employing a random-effects model yielded a pooled incidence of 65.54% (95% CI: 62.58%–68.39%) for ORR (**Figure 2A**). Moderate heterogeneity was noted across the eligible studies (I² = 67.0%, *p* < 0.0001).

**Figure 2:**
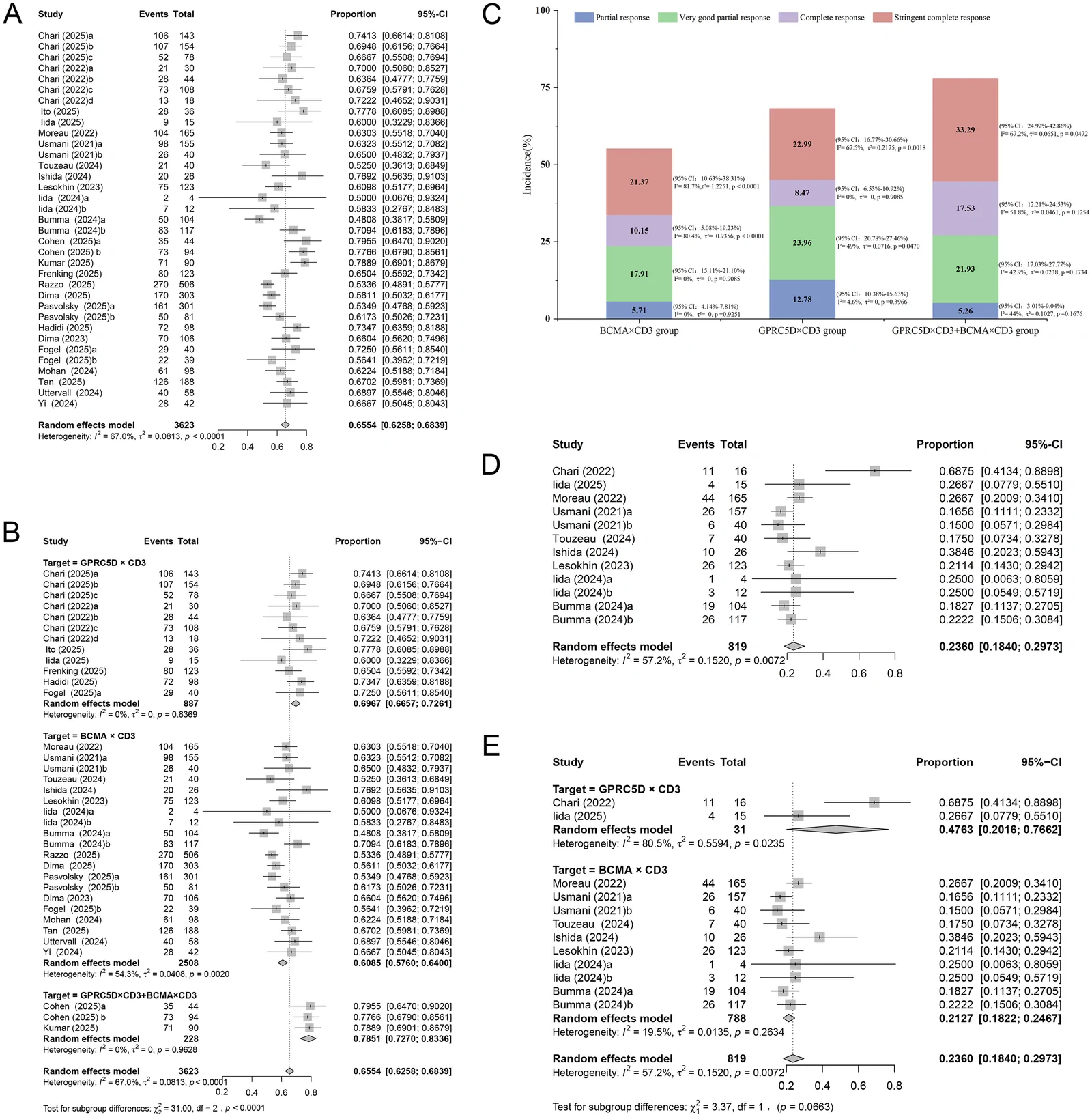
**(A)** Forest plot for pooled overall response rate (ORR) via meta-analysis with the random effects model; **(B)** Forest plot of subgroup analysis for overall response rate (ORR) stratified by therapeutic target using random-effects meta-analysis; **(C)** Pooled incidence of (sCR), (CR), (VGPR), (PR); **(D)** Forest plot of pooled minimal residual disease (MRD) negativity based on random-effects meta-analysis; **(E)** Forest plot of subgroup analysis for minimal residual disease (MRD) negativity stratified by therapeutic target using random-effects meta-analysis.

Subgroup analysis stratified by the target of bispecific antibodies revealed differing pooled incidences of ORR for the GPRC5D×CD3, BCMA×CD3, and GPRC5D×CD3 plus BCMA×CD3 subgroups. Within the GPRC5D×CD3 subgroup (n = 887), the random-effects model yielded a pooled ORR of 69.67% (95% CI: 66.57%–72.61%), with no appreciable heterogeneity detected (I² = 0%, *p* = 0.8369). Within the BCMA×CD3 subgroup (n = 2,508), the pooled ORR was 60.85% (95% CI: 57.60%–64.00%), and moderate heterogeneity was present (I² = 54.3%, *p* = 0.0020). Within the GPRC5D×CD3 plus BCMA×CD3 combination subgroup (n = 228), the pooled ORR was 78.51% (95% CI: 72.70%–83.36%), with no marked heterogeneity observed (I² = 0%, *p* = 0.9628) (**Figure 2B**).

Sensitivity analysis for ORR indicated that the pooled effect estimate was robust and not disproportionately influenced by any single study (**Supplementary Figure 1A**). Assessment of publication bias revealed an asymmetric funnel plot and an Egger’s test *P* of 0.0012 (**Supplementary Figure 1B**). The trim-and-fill method was applied to adjust for publication bias. The corrected pooled effect estimate was 59.89% (95% CI: 56.21%–63.46%) (**Supplementary Figure 1C**), representing only a modest reduction relative to the uncorrected estimate. This suggested that the asymmetry of the funnel plot may reflect an association of study size with underlying response rates, together with clinical heterogeneity, rather than publication bias alone.

#### 3.3.2 Incidence of sCR, CR, VGPR, and PR

As illustrated in **Figure 2C**, the constituent proportions of sCR, CR, VGPR, and PR differed across the three treatment groups. In the BCMA×CD3 group, the pooled incidences of PR, VGPR, CR, and sCR were 5.71% (95% CI: 4.14%–7.81%; I² = 0%), 17.91% (15.11%–21.10%; I² = 0%), 10.15% (5.08%–19.23%; I² = 80.4%), and 21.37% (10.63%–38.31%; I² = 81.7%), respectively. The corresponding incidences in the GPRC5D×CD3 group were 12.78% (10.38%–15.63%; I² = 4.6%), 23.96% (20.78%–27.46%; I² = 49.0%), 8.47% (6.53%–10.92%; I² = 0%), and 22.99% (16.77%–30.66%; I² = 67.5%). In the GPRC5D×CD3 plus BCMA×CD3 combination group, respective incidences of PR, VGPR, CR, and sCR were 5.26% (3.01%–9.04%; I² = 44.0%), 21.93% (17.03%–27.77%; I² = 42.9%), 17.53% (12.21%–24.53%; I² = 51.8%), and 33.29% (24.92%–42.86%; I² = 67.2%). The detailed forest plots of this meta-analysis are presented in **Supplementary Figures 2A–D**.

#### 3.3.3 Incidence of MRD negativity

Across the 12 cohorts encompassing 819 patients with MRD negativity, the random-effects model yielded a pooled MRD-negativity rate of 23.6% (95% CI: 18.4%–29.7%) following treatment with bispecific antibodies. Reported MRD-negativity rates in individual studies ranged from 15.00% to 68.75%, with moderate heterogeneity present across studies (I² = 57.2%, *p* = 0.0072; **Figure 2D**).

Subgroup analysis stratified by target revealed that the GPRC5D×CD3 subgroup, comprising 2 studies and 31 patients, had a pooled MRD-negativity rate of 47.6% (95% CI: 20.2%–76.6%), accompanied by high heterogeneity (I² = 80.5%, *p* = 0.0235). The BCMA×CD3 subgroup, comprising 10 studies and 788 patients, had a pooled MRD-negativity rate of 21.3% (18.2%–24.7%), with low heterogeneity across studies (I² = 19.5%, *p* = 0.2634; **Figure 2E**).

Sensitivity analysis and publication bias: Sensitivity analysis for MRD negativity indicated that the result was robust. Under the random-effects model, the pooled effect estimate for the MRD-negativity rate was 23.60% (95% CI: 18.40–29.73). Upon iterative exclusion of individual studies, the pooled estimates for the remaining studies fluctuated between 21.39% and 24.78%, the 95% CIs for each iteration overlapped with that of the original pooled estimate, and no result deviated markedly from the overall trend. Sensitivity analysis thus confirmed the robustness of the pooled effect, with heterogeneity declining substantially (I² = 12.2%) after removing the study by Chari et al. (2022) (**Supplementary Figure 1D**). Assessment of publication bias revealed a symmetric funnel plot and an Egger’s test *P* of 0.3773, suggesting no significant publication bias (**Supplementary Figure 1E**).

### 3.4 Hematological AEs

#### 3.4.1 Incidence of any-grade hematological AEs

All eligible studies reported any-grade hematological AEs. The pooled analysis demonstrated that, in the BCMA×CD3 group, neutropenia and anemia were the most common events, with pooled incidences of 57.25% (95% CI: 47.38%–66.58%; I² = 90.5%) and 58.59% (95% CI: 47.54%–68.85%; I² = 80.3%), respectively, followed by thrombocytopenia (45.66%; 95% CI: 36.69%–54.92%; I² = 78.9%), leukopenia (39.49%; 95% CI: 24.90%–56.24%; I² = 91.9%), and lymphopenia (28.68%; 95% CI: 20.65%–38.34%; I² = 75.4%; **Figure 3A**).

**Figure 3:**
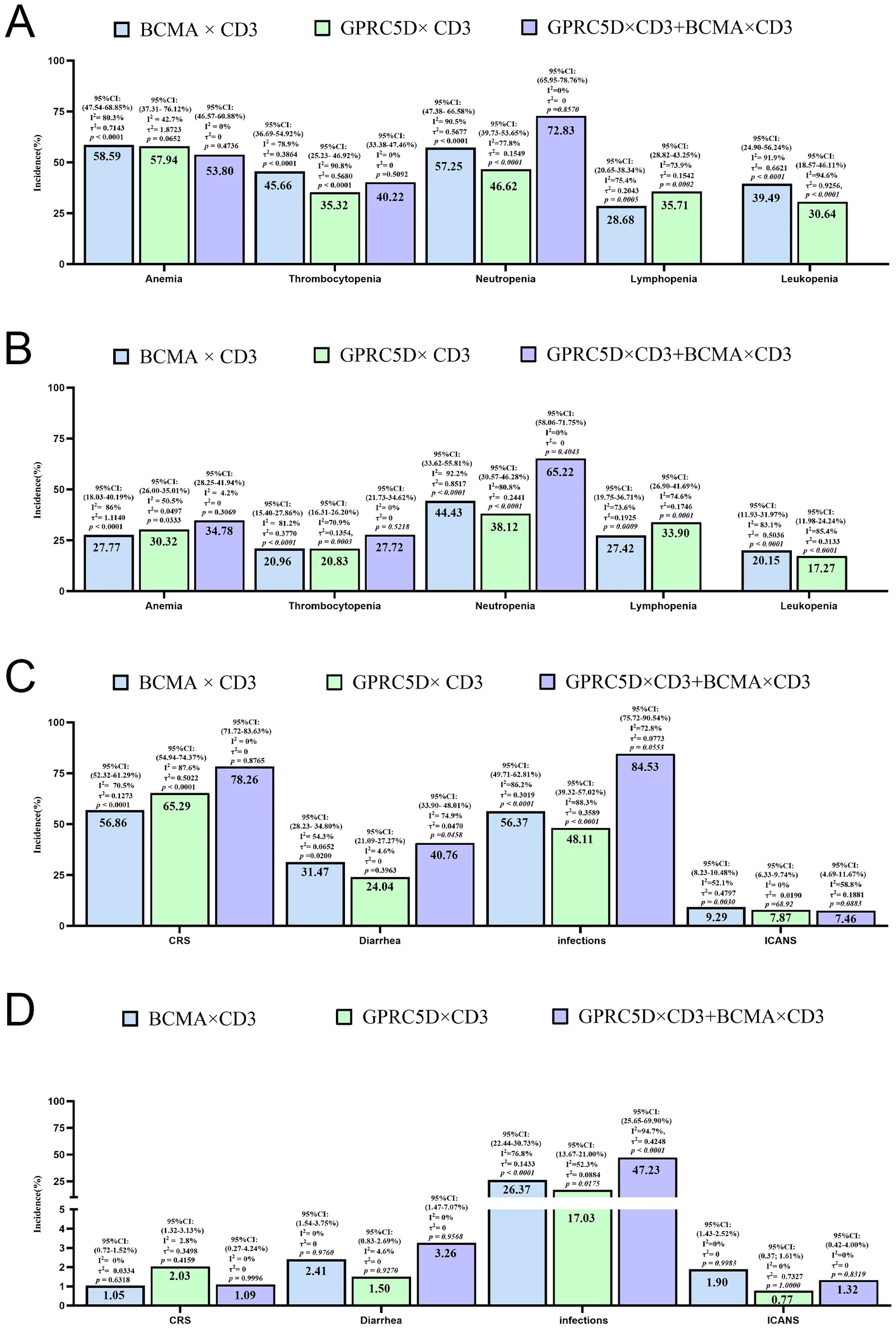
**(A)** Pooled incidence of any-grade hematologic adverse events; **(B)** Pooled incidence of grade 3–4 hematologic adverse events; **(C)** Pooled incidence of any-grade non-hematologic adverse events; **(D)** Pooled incidence of grade 3–4 non-hematologic adverse events.

In the GPRC5D×CD3 group, the pooled incidence was 57.94% for anemia (95% CI: 37.31%–76.12%; I² = 42.7%), 46.62% for neutropenia (39.73%–53.65%; I² = 77.8%), 35.71% for lymphopenia (28.82%–43.25%; I² = 73.9%), 35.32% for thrombocytopenia (25.23%–46.92%; I² = 90.8%), and 30.64% for leukopenia (18.57%–46.11%; I² = 94.6%; **Figure 3A**).

In the GPRC5D×CD3 plus BCMA×CD3 dual-target group, the pooled incidence was 72.83% for neutropenia (95% CI: 65.95%–78.76%; I² = 0%), 53.80% for anemia (46.57%–60.88%; I² = 0%), and 40.22% for thrombocytopenia (33.38%–47.46%; I² = 0%; **Figure 3A**). The detailed forest plots of this meta-analysis are presented in **Supplementary Figures 3A–E**.

#### 3.4.2 Incidence of grade 3–4 hematological AEs

Pooled analysis of grade 3–4 hematological AEs indicated that neutropenia was the most common grade 3–4 hematological toxicity, with respective incidences of 44.43% (95% CI: 33.62%–55.81%), 38.12% (30.57%–46.28%), and 65.22% (58.06%–71.75%) in the BCMA×CD3, GPRC5D×CD3, and GPRC5D×CD3 plus BCMA×CD3 groups. The pooled incidences of anemia were 27.77% (18.03%–40.19%), 30.32% (26.00%–35.01%), and 34.78% (28.25%–41.94%); those of thrombocytopenia were 20.96% (15.40%–27.86%), 20.83% (16.31%–26.20%), and 27.72% (21.73%–34.62%). In the BCMA×CD3 and GPRC5D×CD3 groups, the incidences of lymphopenia were 27.42% (19.75%–36.71%) and 33.90% (26.90%–41.69%), respectively, and those of leukopenia were 20.15% (11.93%–31.97%) and 17.27% (11.98%–24.24%; **Figure 3B**). The detailed forest plots of this meta-analysis are presented in **Supplementary Figures 4A–E**.

### 3.5 Non-hematological AEs

#### 3.5.1 Incidence of any-grade non-hematological AEs

Pooled analysis of any-grade non-hematological AEs showed that CRS was among the common events. Its incidences were 56.86% (95% CI: 52.32%–61.29%), 65.29% (54.94%–74.37%), and 78.26% (71.72%–83.63%) in the BCMA×CD3, GPRC5D×CD3, and GPRC5D×CD3 plus BCMA×CD3 dual-target groups, respectively. The pooled incidences of infection were 56.37% (49.71%–62.81%), 48.11% (39.32%–57.02%), and 84.53% (75.72%–90.54%). The incidence of diarrhea was 31.47% (28.23%–34.80%), 24.04% (21.09%–27.27%), and 40.76% (33.90%–48.01%). The overall incidence of ICANS was low, at 9.29% (8.23%–10.48%), 7.87% (6.33%–9.74%), and 7.46% (4.69%–11.67%), respectively (**Figure 3C**). The detailed forest plots of this meta-analysis are presented in **Supplementary Figures 5A–D**.

#### 3.5.2 Incidence of grade 3–4 non-hematological AEs

Among grade 3–4 non-hematological AEs, severe infection was the most frequent event. Its pooled incidences were 26.37% (95% CI: 22.44%–30.73%), 17.03% (13.67%–21.00%), and 47.23% (25.65%–69.90%) in the BCMA×CD3, GPRC5D×CD3, and GPRC5D×CD3 plus BCMA×CD3 groups, respectively. The incidence of grade 3–4 CRS was low, at 1.05% (0.72%–1.52%), 2.03% (1.32%–3.13%), and 1.09% (0.27%–4.24%). The pooled incidences of grade 3–4 diarrhea were 2.41% (1.54%–3.75%), 1.50% (0.83%–2.69%), and 3.26% (1.47%–7.07%). Grade 3–4 ICANS was uncommon, with pooled incidences of 1.90% (1.43%–2.52%), 0.77% (0.37%–1.61%), and 1.32% (0.42%–4.00%). (**Figure 3D**). The detailed forest plots of this meta-analysis are presented in **Supplementary Figures 6A–D**.

#### 3.5.3 Incidence of grade 1–2 taste-related changes

Across 15 study cohorts, the overall pooled incidence of grade 1–2 taste-related changes was 65.18% (95% CI: 51.61%–76.66%; I² = 87.3%, *p* < 0.0001) (**Supplementary Figure 7A**). Stratified by target, the pooled incidence of treatment-related grade 1–2 taste-related changes was 69.89% (60.74%–77.69%; I² = 81.9%) in the GPRC5D×CD3 group, 77.28% (58.46%–89.15%; I² = 91.0%) in the GPRC5D×CD3 plus BCMA×CD3 combination group, and 16.42% (5.70%–39.00%; I² = 80.7%) in the BCMA×CD3 group (**Figure 4**) (**Supplementary Figure 7B**). Overall, grade 1–2 taste-related changes were concentrated predominantly in GPRC5D-directed and dual-targeted treatment cohorts.

**Figure 4:**
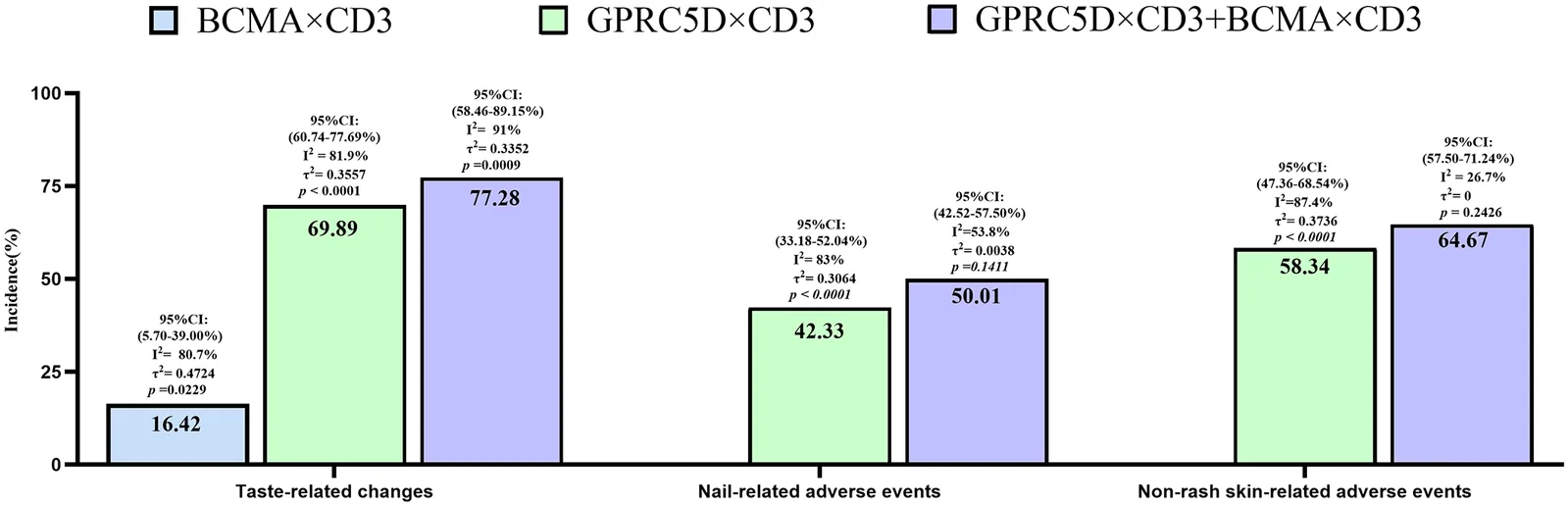
Pooled incidence of any-grade epithelium-related toxicity.

#### 3.5.4 Incidence of any-grade nail-related AEs

In 12 cohorts encompassing 942 patients, the pooled incidence of any-grade nail-related AEs was 43.85% (95% CI: 35.97%–52.05%), with high heterogeneity across studies (I² = 80.4%, *p* < 0.0001) (**Supplementary Figure 7C**). Subgroup analysis stratified by target revealed that the pooled incidence of any-grade nail-related AEs linked to GPRC5D×CD3 bispecific antibodies was 42.33% (33.18%–52.04%; I² = 83.0%), and that in the GPRC5D×CD3 plus BCMA×CD3 combination group was 50.01% (42.52%–57.50%; I² = 53.8%) (**Figure 4**) (**Supplementary Figure 7D**). This AE was not reported in the BCMA×CD3 treatment group.

#### 3.5.5 Incidence of grade 3–4 nail-related AEs

For grade 3–4 nail-related AEs, 12 studies comprising 942 patients were included. Only 2 events were reported in total, and the fixed-effects model yielded a pooled incidence of 0.21% (95% CI: 0.05%–0.84%). The incidence was low in each study, and most investigations found no grade 3–4 events; only Chari et al., 2022b and Chari et al., 2022c each reported a single event. No marked heterogeneity was observed across studies (I² = 0.0%, *p* = 1.0000) (**Supplementary Figure 7E**).

#### 3.5.6 Incidence of any-grade non-rash skin-related AEs

Among the 11 study cohorts encompassing 916 patients, any-grade non-rash skin-related AEs were relatively common. The random-effects model yielded a pooled incidence of 59.7% (95% CI: 50.7%–68.1%). Reported incidences in individual studies ranged widely from 23.5% to 73.4%, with high inter-study heterogeneity (I² = 85.0%, *p* < 0.0001) (**Supplementary Figure 7F**).

According to subgroup analysis stratified by therapeutic target, the GPRC5D×CD3 subgroup exhibited a pooled incidence of any-grade non-rash skin-related AEs of 58.3% (95% CI: 47.4%–68.5%; I² = 87.4%, *p* < 0.0001). In the GPRC5D×CD3 plus BCMA×CD3 combination subgroup, the pooled incidence was 64.7% (57.5%–71.2%), with low heterogeneity (I² = 26.7%, *p* = 0.2426) (**Figure 4**) (**Supplementary Figure 7G**). This AE was not reported in the BCMA×CD3 subgroup.

#### 3.5.7 Incidence of grade 3–4 non-rash skin-related AEs

Among the 11 study cohorts encompassing 916 patients, the overall incidence of grade 3–4 non-rash skin-related AEs was exceedingly low. Only three cohorts reported such events, with the incidences for individual studies ranging from 0% to 2.27%. The fixed-effects model yielded a pooled overall incidence of 0.33% (95% CI: 0.11%–1.01%) for grade 3–4 non-rash skin-related AEs. No statistical heterogeneity was detected across studies (I² = 0.0%, *p* = 0.9999) (**Supplementary Figure 7H**).

### 3.6 Subgroup analysis restricted to real-world studies

The real-world ORR was 60.03% (95% CI: 55.87%–64.05%) for BCMA×CD3 bispecific antibodies and 69.35% (95% CI: 63.49%–74.64%) for GPRC5D×CD3 bispecific antibodies, consistent with the pooled overall estimates. The subgroup difference across therapeutic targets remained statistically significant (*p* = 0.0104) (**Supplementary Figure 8A**).

With respect to hematological toxicity, the pooled incidence of neutropenia was 53.05% (95% CI: 35.66%–69.73%) in the BCMA×CD3 group and 57.56% (95% CI: 50.06%–64.73%) in the GPRC5D×CD3 group (**Supplementary Figure 8B**). The pooled incidence of thrombocytopenia was 51.86% (95% CI: 36.06%–67.30%) for the BCMA×CD3 group versus 64.64% (95% CI: 28.45%–89.37%) for the GPRC5D×CD3 group (**Supplementary Figure 8C**). For anemia, the pooled incidence was 66.53% (95% CI: 47.26%–81.51%) in the BCMA×CD3 group and 98.76% (95% CI: 0.0620–1.0000) in the GPRC5D×CD3 group (**Supplementary Figure 8D**).

In real-world studies, the pooled incidence of infection was 51.53% (95% CI: 43.00%–59.97%) in the BCMA×CD3 group and 40.36% (95% CI: 27.13%–55.15%) in the GPRC5D×CD3 group (**Supplementary Figure 8E**). The pooled incidence of CRS was 53.21% (95% CI: 47.79%–58.55%) for BCMA×CD3 and 47.35% (95% CI: 24.67%–71.17%) for GPRC5D×CD3 (**Supplementary Figure 8F**). The pooled incidence of ICANS was 11.05% (95% CI: 9.71%–12.54%) in the BCMA×CD3 group and 9.09% (95% CI: 6.35%–12.85%) in the GPRC5D×CD3 group (**Supplementary Figure 8G**). The pooled incidence of dysgeusia was 16.42% (95% CI: 5.70%–39.00%) in the BCMA×CD3 group and 79.92% (95% CI: 59.74%–91.43%) in the GPRC5D×CD3 group (**Supplementary Figure 8H**).

## 4 Discussion

The present systematic review and meta-analysis incorporated 27 clinical trials and real-world investigations involving 4,045 patients with RRMM. By synthesizing the available evidence, including data from dual-target trials, we comprehensively profiled the efficacy and antigen-specific toxicity spectra of three bispecific antibody strategies according to target antigen.

At the efficacy level, the pooled ORR of the three strategies exhibited target-stratified differences. The dual-target combination achieved an ORR of 78.51%. The biological basis resides in the simultaneous targeting of BCMA and GPRC5D—two plasma-cell antigens with relatively independent expression—which may suppress immune escape arising from single-antigen loss while augmenting the tumor-killing effect of T-cell redirection (33). This mechanistic rationale is further supported by the most recent update of the RedirecTT-1 programme, in which the phase 2 cohort enrolled 90 patients with drug-resistant, true extramedullary myeloma. In that cohort, talquetamab plus teclistamab produced a response in 79% of patients, with a complete response or better in 54%, a median duration of response of 13.8 months, and 12-month progression-free and overall survival of 61% and 74%, respectively (34). Notably, responses were retained after previous T-cell-redirecting exposure, with a very good partial response or better in 83% of the 18 patients previously treated with anti-BCMA CAR-T therapy and a response in 6 of 8 patients previously exposed to a non-BCMA/non-GPRC5D bispecific antibody (34). These findings indicate that the activity of dual antigen targeting is retained in the biologically most adverse subsets of RRMM. Nevertheless, because the current evidence derives predominantly from the RedirecTT-1 clinical trial with a small sample size, the efficacy of GPRC5D plus BCMA cannot be adequately evaluated. This observation should be regarded as hypothesis-generating and still requires validation in head-to-head randomized studies, and should not be taken as formal clinical recommendations. A recently published meta-analysis of a similar nature (17), although incorporating both BCMA×CD3 and GPRC5D×CD3 antibodies, establishes only two monospecific subgroups and does not evaluate the dual-target combination as an independent category. The dual-target group, while achieving an elevated response rate, also exhibited greater burdens of infection (84.53%) and hematological toxicity. This suggests that its efficacy advantage comes at the cost of superimposed toxicity. The comparatively lower efficacy in the BCMA×CD3 subgroup warrants cautious interpretation because this subgroup included a larger proportion of real-world cohorts; patients in these cohorts typically present with higher disease burden, more comorbidities, and more complex histories of prior treatments, which may partially underestimate the true efficacy. Accordingly, the current comparisons of between-target efficacy should be understood as indirect comparisons based on the available evidence, rather than a definitive ranking of agent or target superiority.

MRD negativity serves as a pivotal endpoint reflecting the depth of remission and correlating closely with long-term prognosis (49). In the target-stratified MRD-negativity analysis, the GPRC5D×CD3 subgroup comprised only two studies with 31 patients. Its 95% CI was extremely wide, and the between-subgroup difference had no statistical significance. Accordingly, this numerical difference should not be interpreted as evidence of inferior depth of remission with BCMA-targeted therapy. The rates of ≥CR (CR plus sCR) were 31.52% and 31.46% for the GPRC5D×CD3 and BCMA×CD3 subgroups, respectively. This implies that the observed disparity in MRD-negativity rates is more likely driven by variations in the availability of MRD testing and denominator definitions rather than genuine differences in depth of treatment response. Half of the cohorts within the BCMA×CD3 subgroup were real-world series, in which MRD assessment was not routinely performed. Furthermore, several of these cohorts specifically enrolled patients previously failing BCMA-directed therapies, more frequent dose reductions were applied, and follow-up durations were relatively short. These factors may systematically underestimate the MRD-negativity rate for BCMA×CD3-based therapy. The heterogeneity declined markedly after excluding Chari et al. (2022) (23). This suggests that the study by Chari et al. (23) constituted the principal source of heterogeneity, likely attributable to its early dose-finding phase, the enrolled population, and differences in detection methods for MRD. Prior meta-analyses of comparable scope have generally adopted clinical response endpoints such as sCR and CR without including MRD (17). This highlights the need for harmonized standards of MRD detection, uniform reporting conventions, and further validation in larger cohort studies.

At the safety level, the most clinically consequential finding is that distinct targets exhibit clearly defined antigen-specific toxicity spectra—precisely the reason why target-stratified analysis, rather than pooled assessment, is warranted. BCMA×CD3-directed therapy is prominently characterized by a substantial burden of infection. The incidences of both any-grade infection (56.37%) and grade 3–4 infection (26.37%) were elevated, accompanied by more pronounced hematological toxicity. The underlying mechanism involves the fact that BCMA is expressed on malignant plasma cells and broadly on normal plasma cells, where it participates in maintaining humoral immunity; targeting BCMA inevitably depletes normal plasma cells, induces profound hypogammaglobulinemia, and delays immune reconstitution. This leads to sustained immunosuppression and a high risk of infection (50). The current target-stratified subgroup results are highly consistent with this biological mechanism and align with those of previous meta-analyses (51). For patients receiving BCMA×CD3-directed therapy, future investigations and clinical practice should systematically report baseline levels of immunoglobulins, utilization of intravenous immunoglobulin (IVIG), antiviral/antibacterial/antifungal prophylaxis strategies, status of vaccination, types of severe infection, and infection-related mortality to more accurately assess the risk of infection and optimize supportive care. The moderate-to-high heterogeneity observed for any-grade infection may stem from disparities among real-world cohorts and clinical trials in baseline risk of infection, dosing regimens, prophylaxis strategies for infection, and use of IVIG. The RedirecTT-1 experience provides a concrete illustration of how modifiable this risk may be. In the phase 1b portion, grade 3 or 4 infections occurred in 63.82% of patients, and only 57% of those with non-IgG myeloma received immunoglobulin replacement (33). In the subsequent phase 2 extramedullary cohort, conducted with protocolized anti-infective management, grade 3 or 4 infections fell to 31.11%, while 87% of patients received at least one dose of IVIG (median 50 days from the first study dose), 97% received antiherpes prophylaxis, and 82% were vaccinated against COVID-19 (34). In both cohorts, severe infections clustered within the first 6 months of therapy and declined thereafter. Of note, in the phase 2 cohort, three of the five patients who died of infection had not received IVIG (34), underscoring that infection-related mortality with T-cell-redirecting therapy is at least partly preventable.

In contrast, GPRC5D×CD3-directed therapy manifests a characteristic toxicity spectrum dominated by epithelium-related toxicity—an innovative dimension that the present analysis has deliberately characterized and that prior meta-analyses (17, 51) do not systematically present. This toxicity profile has a clear histological basis. GPRC5D is expressed on malignant plasma cells and widely distributed in keratinized tissues, including filiform papillae of the dorsal tongue, epithelium of taste buds, the basal layer of hair follicles, and the nail bed. Local T-cell activation mediated by the bispecific antibody disrupts these microenvironments dependent on rapid turnover, giving rise to taste-related changes, impaired mucosal repair, and compromised integrity of the keratinized barrier (52). The target-stratified subgroup results indicate that the incidences of grade 1–2 taste-related changes, any-grade nail-related events, and any-grade non-rash skin-related events are broadly comparable between the GPRC5D×CD3 group and the GPRC5D×CD3 plus BCMA×CD3 combination group. Consistent with this, the RedirecTT-1 phase 2 cohort reported oral AEs in 87% of patients, non-rash skin events in 69%, and nail-related events in 56%, yet only four oral events reached grade 3 or 4 and no non-rash skin or nail event exceeded grade 2. Talquetamab was discontinued for oral or skin toxicity in only two patients (34). This indicates that adding a BCMA-directed agent does not qualitatively amplify GPRC5D-associated epithelial toxicity, which remains predominantly low-grade but pervasive. The BCMA×CD3 group, by contrast, rarely reports such events. Notably, although the incidences of these events are high, severe events are exceedingly rare. The pooled incidences of grade 3–4 nail-related and non-rash skin-related AEs stand at merely 0.21% and 0.33%, respectively. GPRC5D-related epithelial toxicity should not be simplistically dismissed as mild toxicity. Although high-grade events are rare, taste-related changes, xerosis, desquamation, and nail disorders are chronic and cumulative, affecting quality of life, nutritional intake, weight maintenance, treatment adherence, and the sustainability of long-term administration. This analysis, therefore, suggests that safety evaluation of GPRC5D-targeted agents should systematically incorporate patient-reported outcomes, taste- and nutrition-related indices, dermatological and nail-specific assessments, and indicators of treatment persistence. The high heterogeneity noted for these epithelial toxicity outcomes may reflect inconsistent definitions and methods of active ascertainment for taste, skin, and nail events across studies, along with the fact that a majority of BCMA-directed studies do not systematically report such events.

The results of stratified analysis for CRS and ICANS across the three targeting strategies suggest that immune-mediated acute toxicities are generally manageable. CRS was relatively common in all three groups but was predominantly low-grade, and the incidence of grade 3–4 CRS was minimal; the overall incidence of ICANS was low, and severe ICANS was rare. These findings imply that current management strategies—including step-up dosing, inpatient monitoring, and early administration of tocilizumab and corticosteroids—may have substantially mitigated the risk of severe immune-mediated toxicity (53). This observation carries relevance for clinical decision-making when weighing bispecific antibodies against CAR T-cell therapy. A schematic overview of the mechanisms and key findings from the present study is provided in **Figure 5**.

**Figure 5:**
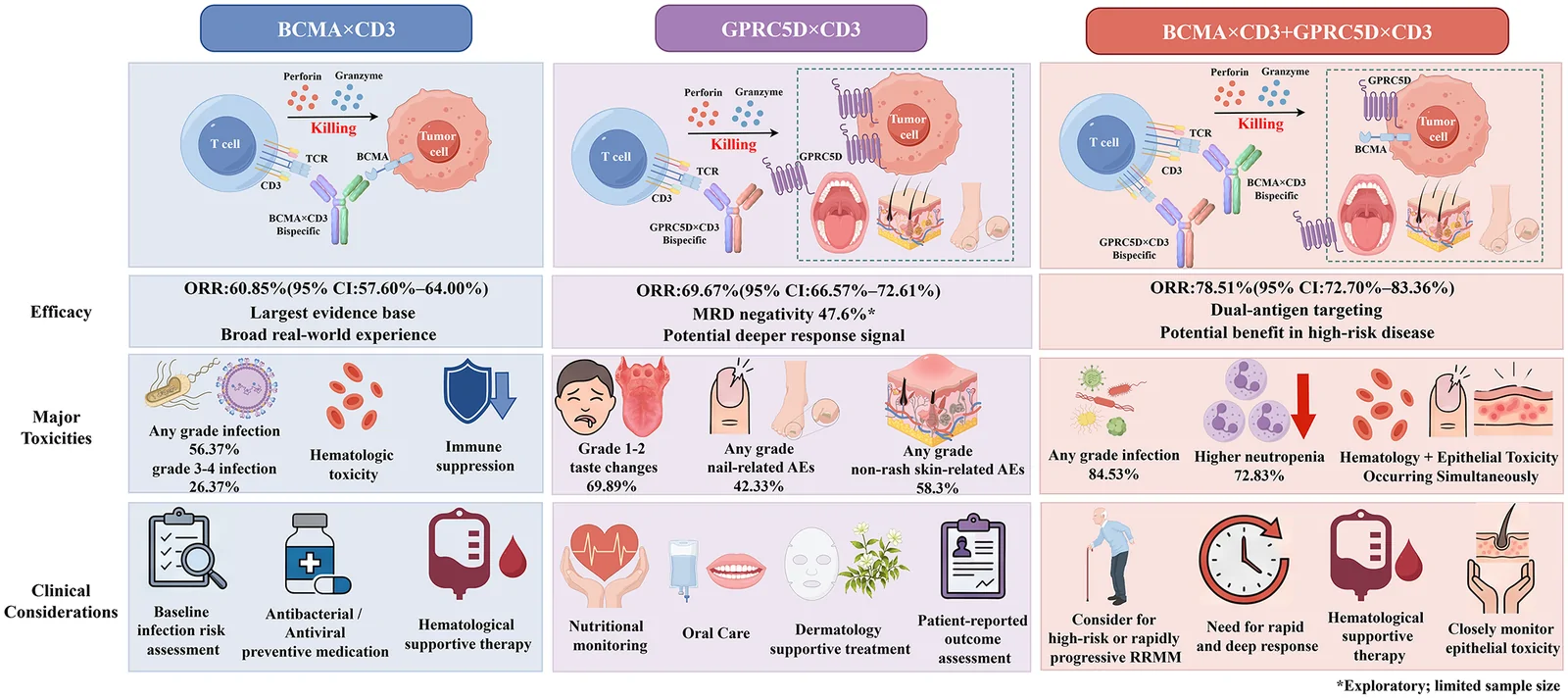
Schematic overview comparing efficacy, major toxicities, and clinical considerations for BCMA×CD3, GPRC5D×CD3, and BCMA×CD3 plus GPRC5D×CD3 bispecific antibody therapies. (By Figdraw).

### 4.1 Limitations

Several limitations should be considered. First, all eligible studies employed single-arm, open-label designs, and direct head-to-head randomized comparisons are lacking; between-target differences arise primarily from indirect comparisons. In particular, the BCMA subgroup incorporated a larger number of real-world cohorts, which bore a greater burden of baseline disease and had undergone more extensive prior treatment, possibly underestimating the relative efficacy. Second, moderate-to-high heterogeneity was present for multiple outcomes. It reflects disparities in enrollment criteria, dosing schedules, and reporting standards across early dose-finding studies, pivotal phase II trials, and real-world investigations. Third, the definitions and methods of active ascertainment for taste, skin, and nail events were inconsistent across studies, and some BCMA-directed studies did not systematically report these events, which may influence the accuracy of the between-subgroup comparisons for epithelial toxicity. Fourth, the dual-target combination subgroup does not constitute an independent multi-study pooled estimate, and the subgroup analyses of MRD are constrained by small sample sizes and wide CIs. The corresponding conclusions should be interpreted cautiously. Fifth, as the present investigation relied on study-level data, it could not adjust for individual-level factors, including age, renal function, extramedullary disease, high-risk cytogenetics, and prior exposure to BCMA- or T-cell-redirecting therapies.

### 4.2 Clinical implications and future directions

The target-stratified results integrating efficacy and toxicity carry implications for the individualized treatment selection of patients with RRMM. For patients with a history of recurrent infections, pronounced hypogammaglobulinemia, poor bone marrow reserve, or elevated baseline infection risk, intensified prophylactic management strategies should be adopted for BCMA×CD3-directed therapy. This includes front-loaded and intensified monitoring during the first 6 months; systematic surveillance of IgG levels with pre-emptive rather than reactive immunoglobulin replacement, particularly when IgG is below 400 mg/dL; and standardized antiviral and Pneumocystis jirovecii prophylaxis (33, 34). Conversely, for patients in whom preserving nutritional status, quality of life, and long-term treatment adherence is a priority, the risks of taste, skin, and nail-related toxicity should be fully communicated before initiating GPRC5D×CD3-directed therapy. Supportive measures include pretreatment oral assessment, regular use of a soft toothbrush and non-irritating mouth rinses, fragrance-free emollients for xerosis, early dermatological evaluation for persistent rash or nail changes, and dietary counselling with regular monitoring of body weight and oral intake when taste disturbances occur. The optimal use of dual-target therapy combining GPRC5D×CD3 and BCMA×CD3 will depend on identifying patient populations in whom the expected efficacy benefit outweighs the risk of cumulative toxicity. Treatment may be optimized through sequential therapy, intermittent dosing, or risk-adapted dose strategies.

First, future investigations should pursue head-to-head randomized controlled trials and meta-analyses of individual patient data to more reliably compare the efficacy and toxicity of distinct targets and dual-target strategies. Second, consensus criteria for standardized documentation of target-specific AEs are urgently required. Taste, oral, dermatological, and nail events are currently captured with inconsistent terminology, variable grouping of preferred terms, and largely passive ascertainment, which limits comparability across studies. Third, uniform standards of MRD assessment are required, including unified sensitivity thresholds (10^-5^ vs. 10^-6^), harmonized next-generation sequencing and next-generation flow detection platforms, predefined assessment time points, and consistent reporting of sustained MRD negativity. Fourth, long-term effectiveness should be addressed through pooled analyses of real-world cohorts and registries reporting progression-free survival, overall survival, time to next treatment, and duration of response. Fifth, the mechanisms of cross-resistance following different targeted therapies should be further clarified to provide a basis for optimizing the treatment sequencing of RRMM. Sixth, prospective studies should evaluate optimal dose intensity, response-adapted or de-escalated schedules, outpatient step-up administration, infection-prophylaxis and immunoglobulin-replacement algorithms, and outcomes in older or frail patients and in those with renal impairment or extramedullary disease, alongside quality-of-life and health-economic endpoints.

## 5 Conclusion

The current systematic review and meta-analysis demonstrates that the efficacy and safety of bispecific antibodies in RRMM exhibit a marked target-dependent pattern. BCMA×CD3-directed therapy is principally related to risks of infection and myelosuppression, whereas GPRC5D×CD3-directed therapy is linked to taste, nail, and non-rash skin-related toxicities, with severe epithelial events remaining rare. These results support target-stratified, individualized selection of bispecific antibodies in RRMM based on patients’ risk of infection, bone marrow reserve, quality-of-life priorities, and treatment goals. Head-to-head randomized trials and harmonized standards for MRD and assessment of epithelial toxicity are still required to validate these findings.

## Data Availability

The original contributions presented in the study are included in the article/**Supplementary Material**. Further inquiries can be directed to the corresponding author.

## Supplementary material

Supplementary Table 1 Search result records.

Supplementary Table 2 Baseline table of 13 single-arm clinical trials.

Supplementary Table 3 Baseline table of 14 real-world data items.

Supplementary Table 4 MINORS quality evaluation of included studies in this meta-analysis

Supplementary Figure 1 **(A)** Sensitivity analysis for overall response rate (ORR); **(B)** Funnel plot to assess publication bias of the pooled overall response rate (ORR); **(C)** Forest plot generated via the trim-and-fill method to adjust publication bias for pooled overall response rate (ORR); **(D)** Sensitivity analysis for minimal residual disease (MRD) negativity; **(E)** Funnel plot for evaluating publication bias of pooled minimal residual disease (MRD) negativity rate.

Supplementary Figure 2 **(A)** Common effect model meta-analysis forest plot of partial response (PR) subgroup analysis stratified by therapeutic target; **(B)** Forest plot of subgroup analysis stratified by therapeutic target for very good partial response (VGPR) based on meta-analysis with the common effect model; **(C)** Forest plot of subgroup analysis stratified by therapeutic target for complete response (CR) via meta-analysis with the random effects model; **(D)** Forest plot of subgroup analysis stratified by therapeutic target for stringent complete response (sCR) based on meta-analysis with random effects model.

Supplementary Figure 3 **(A)** Forest plot of subgroup analysis stratified by therapeutic target for any-grade anemia from meta-analysis with the random effects model; **(B)** Forest plot of subgroup analysis stratified by therapeutic target for any-grade thrombocytopenia based on meta-analysis with the random effects model; **(C)** Forest plot of subgroup analysis stratified by therapeutic target for any-grade neutropenia based on meta-analysis with the random effects model; **(D)** Forest plot of subgroup analysis stratified by therapeutic target for any-grade lymphopenia based on meta-analysis with the random effects model; **(E)** Forest plot of subgroup analysis stratified by therapeutic target for any-grade leukopenia based on meta-analysis with the random effects model.

Supplementary Figure 4 **(A)** Forest plot of subgroup analysis stratified by therapeutic target for grade 3–4 anemia based on meta-analysis with the random effects model; **(B)** Forest plot of subgroup analysis stratified by therapeutic target for grade 3–4 thrombocytopenia based on meta-analysis with the random effects model; **(C)** Forest plot of subgroup analysis stratified by therapeutic target for grade 3–4 neutropenia based on meta-analysis with the random effects model; **(D)** Forest plot of subgroup analysis stratified by therapeutic target for grade 3–4 lymphopenia based on meta-analysis with the random effects model; **(E)** Forest plot of subgroup analysis stratified by therapeutic target for grade 3–4 leukopenia based on meta-analysis with the random effects model.

Supplementary Figure 5 **(A)** Forest plot of subgroup analysis stratified by therapeutic target for any-grade cytokine release syndrome (CRS) based on meta-analysis with the random effects model; **(B)** Forest plot of subgroup analysis stratified by therapeutic target for any-grade diarrhea based on meta-analysis with the common effect model; **(C)** Forest plot of subgroup analysis stratified by therapeutic target for any-grade infection based on meta-analysis with the random effects model; **(D)** Forest plot of subgroup analysis stratified by therapeutic target for any-grade immune effector cell-associated neurotoxicity syndrome (ICANS) based on meta-analysis with the common effect model.

Supplementary Figure 6 **(A)** Forest plot of subgroup analysis stratified by therapeutic target for grade 3–4 cytokine release syndrome (CRS) based on meta-analysis with the common effect model; **(B)** Forest plot of subgroup analysis stratified by therapeutic target for grade 3–4 diarrhea based on meta-analysis with the common effect model; **(C)** Forest plot of subgroup analysis stratified by therapeutic target for grade 3–4 infection based on meta-analysis with the random effects model; **(D)** Forest plot of subgroup analysis stratified by therapeutic target for grade 3–4 immune effector cell-associated neurotoxicity syndrome (ICANS) based on meta-analysis with the common effect model.

Supplementary Figure 7 **(A)** Forest plot of pooled grade 1–2 taste-related adverse events derived from random-effects meta-analysis; **(B)** Forest plot of subgroup analysis for grade 1–2 taste-related adverse events stratified by therapeutic target using random-effects meta-analysis; **(C)** Forest plot of pooled any-grade nail-related adverse events based on random-effects meta-analysis; **(D)** Forest plot of subgroup analyses for any-grade nail-related adverse events stratified by therapeutic target based on random-effects meta-analysis; **(E)** Forest plot of pooled grade 3–4 nail-related adverse events derived from fixed-effects meta-analysis; **(F)** Forest plot of pooled any-grade non-rash cutaneous adverse events based on random-effects meta-analysis; **(G)** Forest plot of subgroup analyses for any-grade non-rash cutaneous adverse events stratified by therapeutic target using random-effects meta-analysis; **(H)** Forest plot of pooled grade 3–4 non-rash cutaneous adverse events derived from fixed-effects meta-analysis.

Supplementary Figure 8 Forest plots of subgroup analyses stratified by therapeutic target limited to real-world evidence studies. **(A)** Overall response rate (ORR), meta-analysis with the random-effects model; **(B)** Neutropenia, meta-analysis with the random-effects model; **(C)** Thrombocytopenia, meta-analysis with the random-effects model; **(D)** Anemia, meta-analysis with the random-effects model; **(E)** Infection, meta-analysis with the random-effects model; **(F)** Cytokine release syndrome (CRS), meta-analysis with the random-effects model; **(G)** Immune effector cell-associated neurotoxicity syndrome (ICANS), meta-analysis with the common-effect model; **(H)** Dysgeusia, meta-analysis with the random-effects model.
