# Supplementary material for: Efficacy and specific toxicity profiles of GPRC5D- versus BCMA-targeted bispecific antibodies in relapsed/refractory multiple myeloma: a meta-analysis of clinical trials and real-world studies
Source: Front Immunol. 2026 Sep 29;17:1937154. doi: 10.3389/fimmu.2026.1937154 (PMC13658498; doi:10.3389/fimmu.2026.1937154)
Supplement: Supplementary file 1 [file SupplementaryFile1.docx]

**Supplementary Figures**

| **A** |  | **B** |  |
| --- | --- | --- | --- |
| **C** |  | **D** |  |
| **E** |  |  |  |

**Figure S1 (A)** Sensitivity analysis for overall response rate (ORR)**; (B)** Funnel plot to assess publication bias of the pooled overall response rate (ORR)**; (C)** Forest plot generated via the trim-and-fill method to adjust publication bias for pooled overall response rate (ORR)**; (D)** Sensitivity analysis for minimal residual disease (MRD) negativity**; (E)** Funnel plot for evaluating publication bias of pooled minimal residual disease (MRD) negativity rate.

| **A** |  | **B** |  |
| --- | --- | --- | --- |
| **C** |  | **D** |  |

**Fig. S2 (A)** Common effect model meta-analysis forest plot of partial response (PR) subgroup analysis stratified by therapeutic target**; (B)** Forest plot of subgroup analysis stratified by therapeutic target for very good partial response (VGPR) based on meta-analysis with the common effect model; **(C)** Forest plot of subgroup analysis stratified by therapeutic target for complete response (CR) via meta-analysis with the random effects model**; (D)** Forest plot of subgroup analysis stratified by therapeutic target for stringent complete response (sCR) based on meta-analysis with random effects model.

| **A** |  | **B** |  |
| --- | --- | --- | --- |
| **C** |  | **D** |  |
| **E** |  |  |  |

**Fig. S3 (A)** Forest plot of subgroup analysis stratified by therapeutic target for any-grade anemia from meta-analysis with the random effects model**; (B)** Forest plot of subgroup analysis stratified by therapeutic target for any-grade thrombocytopenia based on meta-analysis with the random effects model**; (C)** Forest plot of subgroup analysis stratified by therapeutic target for any-grade neutropenia based on meta-analysis with the random effects model**; (D)** Forest plot of subgroup analysis stratified by therapeutic target for any-grade lymphopenia based on meta-analysis with the random effects model**; (E)** Forest plot of subgroup analysis stratified by therapeutic target for any-grade leukopenia based on meta-analysis with the random effects model.

| **A** |  | **B** |  |
| --- | --- | --- | --- |
| **C** |  | **D** |  |
| **E** |  |  |  |

**Fig. S4 (A)** Forest plot of subgroup analysis stratified by therapeutic target for grade 3–4 anemia based on meta-analysis with the random effects model**; (B)** Forest plot of subgroup analysis stratified by therapeutic target for grade 3–4 thrombocytopenia based on meta-analysis with the random effects model**; (C)** Forest plot of subgroup analysis stratified by therapeutic target for grade 3–4 neutropenia based on meta-analysis with the random effects model**; (D)** Forest plot of subgroup analysis stratified by therapeutic target for grade 3–4 lymphopenia based on meta-analysis with the random effects model**; (E)** Forest plot of subgroup analysis stratified by therapeutic target for grade 3–4 leukopenia based on meta-analysis with the random effects model.

| **A** |  | **B** |  |
| --- | --- | --- | --- |
| **C** |  | **D** |  |

**Fig. S5 (A)** Forest plot of subgroup analysis stratified by therapeutic target for any-grade cytokine release syndrome (CRS) based on meta-analysis with the random effects model**; (B)** Forest plot of subgroup analysis stratified by therapeutic target for any-grade diarrhea based on meta-analysis with the common effect model**; (C)** Forest plot of subgroup analysis stratified by therapeutic target for any-grade infection based on meta-analysis with the random effects model**; (D)** Forest plot of subgroup analysis stratified by therapeutic target for any-grade immune effector cell-associated neurotoxicity syndrome (ICANS) based on meta-analysis with the common effect model.

| **A** |  | **B** |  |
| --- | --- | --- | --- |
| **C** |  | **D** |  |

**Fig. S6 (A)** Forest plot of subgroup analysis stratified by therapeutic target for grade 3–4 cytokine release syndrome (CRS) based on meta-analysis with the common effect model**; (B)** Forest plot of subgroup analysis stratified by therapeutic target for grade 3–4 diarrhea based on meta-analysis with the common effect model**; (C)** Forest plot of subgroup analysis stratified by therapeutic target for grade 3–4 infection based on meta-analysis with the random effects model**; (D)** Forest plot of subgroup analysis stratified by therapeutic target for grade 3–4 immune effector cell-associated neurotoxicity syndrome (ICANS) based on meta-analysis with the common effect model.

**Fig. S7 (A)** Forest plot of pooled grade 1–2 taste-related adverse events derived from random-effects meta-analysis; **(B)** Forest plot of subgroup analysis for grade 1–2 taste-related adverse events stratified by therapeutic target using random-effects meta-analysis; **(C)** Forest plot of pooled any-grade nail-related adverse events based on random-effects meta-analysis; **(D)** Forest plot of subgroup analyses for any-grade nail-related adverse events stratified by therapeutic target based on random-effects meta-analysis; **(E)** Forest plot of pooled grade 3–4 nail-related adverse events derived from fixed-effects meta-analysis; **(F)** Forest plot of pooled any-grade non-rash cutaneous adverse events based on random-effects meta-analysis; **(G)** Forest plot of subgroup analyses for any-grade non-rash cutaneous adverse events stratified by therapeutic target using random-effects meta-analysis; **(H)** Forest plot of pooled grade 3–4 non-rash cutaneous adverse events derived from random-effects meta-analysis.

**Fig. S8** Forest plots of subgroup analyses stratified by therapeutic target limited to real-world evidence studies. **(A)** Overall response rate (ORR), meta-analysis with the random-effects model; **(B)** Neutropenia, meta-analysis with the random-effects model; **(C)** Thrombocytopenia, meta-analysis with the random-effects model; **(D)** Anemia, meta-analysis with the random-effects model; **(E)** Infection, meta-analysis with the random-effects model; **(F)** Cytokine release syndrome (CRS), meta-analysis with the random-effects model; **(G)** Immune effector cell‑associated neurotoxicity syndrome (ICANS), meta-analysis with the common-effect model; **(H)** Dysgeusia, meta-analysis with the random-effects model.
