# Supplementary material for: Efficacy and specific toxicity profiles of GPRC5D- versus BCMA-targeted bispecific antibodies in relapsed/refractory multiple myeloma: a meta-analysis of clinical trials and real-world studies
Source: Front Immunol. 2026 Sep 29;17:1937154. doi: 10.3389/fimmu.2026.1937154 (PMC13658498; doi:10.3389/fimmu.2026.1937154)
Supplement: Supplementary file 2 [file Table1.docx]

**Table S1** Search result records.

| Pubmed | | | |
| --- | --- | --- | --- |
| # | Query | Results | |
| #1 | Search: Multiple Myeloma[MeSH Terms] | 51890 | |
| #2 | Search: "Plasma-Cell Myeloma"OR"Myelomatosis"OR"Myelomatoses"OR"Myeloma-Multiple"OR"myeloma multiplex"OR"Multiple Myeloma"OR"morbus kahler"OR"Kahler Disease"[Title/Abstract] | 69045 | |
| #3 | Search: B-Cell Maturation Antigen[MeSH Terms] | 949 | |
| #4 | Search: "BCMA"OR"B-Cell Maturation Antigen"OR"B cell maturation protein"OR"Teclistamab"OR"Elranatamab"OR"Linvoseltamab"OR"ABBV-383"OR"Alnuctamab"[Title/Abstract] | 2481 | |
| #5 | Search: Antibodies, Bispecific[MeSH Terms] | 5411 | |
| #6 | Search: "GPRC5D"OR"Talquetamab"OR"bispecific antibodies"OR"Bispecific Monoclonal Antibodies"OR"bispecific immunoglobulin"OR"bispecific antibody"OR"Bispecific Antibodies"OR"Bifunctional Antibodies"OR"Forimtamig"OR"LBL-034"[Title/Abstract] | 9476 | |
| #7 | Search: (#1 OR #2)AND (#3 OR #4 OR #5 OR #6) | 5090 | |
| Embase | | | |
| # | Query | Results | |
| #1 | 'multiple myeloma'/exp OR 'multiple myeloma' | 136908 | |
| #2 | 'plasma-cell myeloma':ti,ab,kw OR 'myelomatosis':ti,ab,kw OR 'myelomatoses':ti,ab,kw OR 'myeloma-multiple':ti,ab,kw OR 'myeloma multiplex':ti,ab,kw OR 'multiple myeloma':ti,ab,kw OR 'morbus kahler':ti,ab,kw OR 'kahler disease':ti,ab,kw | 105657 | |
| #3 | 'b cell maturation antigen'/exp | 5583 | |
| #4 | 'bcma':ti,ab,kw OR 'b-cell maturation antigen':ti,ab,kw OR 'b cell maturation protein':ti,ab,kw OR 'teclistamab':ti,ab,kw OR 'elranatamab':ti,ab,kw OR 'linvoseltamab':ti,ab,kw OR 'abbv-383':ti,ab,kw OR 'alnuctamab':ti,ab,kw | 6548 | |
| #5 | 'bispecific antibody'/exp | 10969 | |
| #6 | 'gprc5d':ti,ab,kw OR 'talquetamab':ti,ab,kw OR 'bispecific monoclonal antibodies':ti,ab,kw OR 'bispecific immunoglobulin':ti,ab,kw OR 'bispecific antibody':ti,ab,kw OR 'bispecific antibodies':ti,ab,kw OR 'bifunctional antibodies':ti,ab,kw OR 'forimtamig':ti,ab,kw OR 'lbl-034':ti,ab,kw | 12512 | |
| #7 | (#1 OR #2) AND (#3 OR #4 OR #5 OR #6) | 6210 | |
| Cochrane Library | | | |
| # | Query | Results | |
| #1 | MeSH descriptor: [Multiple Myeloma] explode all trees | 2556 | |
| #2 | (‘Plasma-Cell Myeloma’ OR ‘Myelomatosis’ OR ‘Myelomatoses’ OR ‘Myeloma-Multiple’ OR ‘Multiple Myeloma’ OR ‘Kahler Disease’):ti,ab,kw | 7270 | |
| #3 | MeSH descriptor: [B-Cell Maturation Antigen] explode all trees | 8 | |
| #4 | (‘BCMA’ OR ‘B-Cell Maturation Antigen’ OR ‘B cell maturation protein’ OR ‘Teclistamab’ OR ‘Elranatamab’ OR ’Linvoseltamab’ OR ’ABBV-383’ OR ’Alnuctamab’ ):ti,ab,kw | 378 | |
| #5 | MeSH descriptor: [Antibodies, Bispecific] explode all trees | 148 | |
| #6 | (’GPRC5D’ OR ‘Talquetamab’ OR ’bispecific antibodies’ OR ‘Bispecific Monoclonal Antibodies’ OR ‘bispecific immunoglobulin’ OR ‘bispecific antibody’ OR ‘Bispecific Antibodies’ OR ‘Bifunctional Antibodies’ OR ‘Forimtamig’ OR ‘LBL-034‘):ti,ab,kw | 720 | |
| #7 | (#1 OR #2) AND (#3 OR #4 OR #5 OR #6) | 299 | |
| Web of Science | | | |
| Query | | | Results |
| ”Plasma-Cell Myeloma”OR”Myelomatosis”OR”Myelomatoses”OR”Myeloma-Multiple”OR”myeloma multiplex”OR”Multiple Myeloma”OR”morbus kahler”OR”Kahler Disease” (Topic) | | | 88682 |
| “BCMA” OR “B-Cell Maturation Antigen” OR “B cell maturation protein” OR “Teclistamab” OR “Elranatamab”OR”Linvoseltamab”OR”ABBV-383”OR”Alnuctamab”OR”GPRC5D” OR “Talquetamab”OR”bispecific antibodies” OR “Bispecific Monoclonal Antibodies” OR “bispecific immunoglobulin” OR “bispecific antibody” OR “Bispecific Antibodies” OR “Bifunctional Antibodies” OR “Forimtamig” OR “LBL-034" (Topic) | | | 12649 |
| #1 AND #2 | | | 3206 |
| Web of Science | | | |
| Query | | | Results |
| ”Plasma-Cell Myeloma”OR”Myelomatosis”OR”Myelomatoses”OR”Myeloma-Multiple”OR”myeloma multiplex”OR”Multiple Myeloma”OR”morbus kahler”OR”Kahler Disease” (Topic) | | | 88682 |
| “BCMA” OR “B-Cell Maturation Antigen” OR “B cell maturation protein” OR “Teclistamab” OR “Elranatamab”OR”Linvoseltamab”OR”ABBV-383”OR”Alnuctamab”OR”GPRC5D” OR “Talquetamab”OR”bispecific antibodies” OR “Bispecific Monoclonal Antibodies” OR “bispecific immunoglobulin” OR “bispecific antibody” OR “Bispecific Antibodies” OR “Bifunctional Antibodies” OR “Forimtamig” OR “LBL-034" (Topic) | | | 12649 |
| #1 AND #2 | | | 3206 |
